# Higher-order structural characterisation of native proteins and complexes by top-down mass spectrometry

**DOI:** 10.1039/d0sc04392c

**Published:** 2020-10-20

**Authors:** Mowei Zhou, Carter Lantz, Kyle A. Brown, Ying Ge, Ljiljana Paša-Tolić, Joseph A. Loo, Frederik Lermyte

**Affiliations:** Environmental Molecular Sciences Laboratory, Pacific Northwest National Laboratory Richland WA 99354 USA; Department of Chemistry and Biochemistry, Department of Biological Chemistry, University of California-Los Angeles Los Angeles CA 90095 USA; Department of Chemistry, University of Wisconsin–Madison Madison WI 53706 USA; Department of Cell and Regenerative Biology, University of Wisconsin–Madison Madison WI 53706 USA; Department of Chemistry, Institute of Chemistry and Biochemistry, Technical University of Darmstadt 64287 Darmstadt Germany frederik.lermyte@tu-darmstadt.de; Mass Spectrometry Laboratory, MolSys Research Unit, University of Liège 4000 Liège Belgium; School of Engineering, University of Warwick Coventry CV4 7AL UK

## Abstract

In biology, it can be argued that if the genome contains the script for a cell's life cycle, then the proteome constitutes an ensemble cast of actors that brings these instructions to life. Their interactions with each other, co-factors, ligands, substrates, and so on, are key to understanding nearly any biological process. Mass spectrometry is well established as the method of choice to determine protein primary structure and location of post-translational modifications. In recent years, top-down fragmentation of intact proteins has been increasingly combined with ionisation of noncovalent assemblies under non-denaturing conditions, *i.e.*, native mass spectrometry. Sequence, post-translational modifications, ligand/metal binding, protein folding, and complex stoichiometry can thus all be probed directly. Here, we review recent developments in this new and exciting field of research. While this work is written primarily from a mass spectrometry perspective, it is targeted to all bioanalytical scientists who are interested in applying these methods to their own biochemistry and chemical biology research.

## Abstract 2

Top-down mass spectrometry techniques break up native proteins and complexes to reveal all levels of structural information.

## Graphical abstract

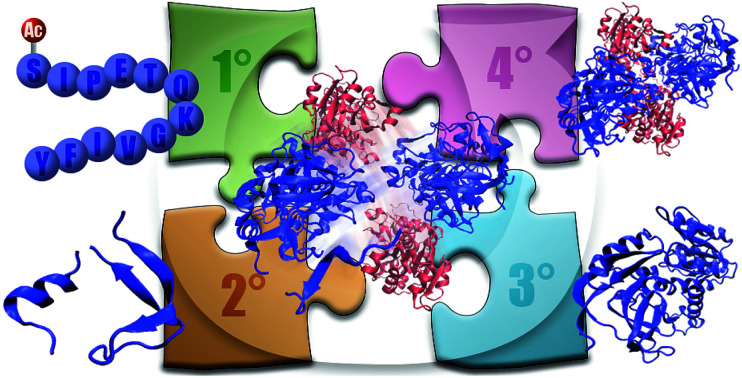

## 1. Introduction and historical perspective

Proteins are the main effectors of biological change; therefore, it is critical to assign their function and dysfunction in cells. In the early 2000s, the human genome was sequenced, leading to the identification of ∼20 000 genes, which might seem like a relatively low number when considering our biological complexity.^1^ The post-genomic era has focused on understanding the downstream diversity that arises as DNA is transcribed into mRNA and then translated into proteins.

At the DNA level, mutations and single-nucleotide polymorphisms represent a substantial source of variation.^2^ During the process of transcription, about 93% of human genes undergo alternative splicing, resulting in variations at the mRNA level.^2^ Further diversity can be introduced after mRNA is translated, with various post-translational modifications (PTMs) occurring to the proteins.^2^ PTMs are not directly encoded in the genome, and many of them are dynamically regulated in response to environmental stress. Combined, DNA, mRNA, and protein-level variations give rise to a diverse set of molecular forms that derive from a single gene. In 2013, a single term, ‘proteoform’,^1,3^ was adopted to clarify the nomenclature surrounding protein complexity and to promote basic and clinical research efforts towards developing technologies for proteoform characterisation.^1^ The total number of human proteoforms has been estimated to be in the hundreds of thousands or even millions.^2^ Mass spectrometry (MS) has emerged as the most versatile and comprehensive method for proteoform characterisation.^4^ Proteins form various noncovalent complexes to perform their biological functions, further complicating the proteome landscape beyond what has been traditionally understood under the proteoform concept. Although MS has mainly been used to obtain information regarding protein sequence, it has been increasingly utilized to understand higher-order structure.^5^

Characterisation of large biomolecules by MS was made possible by soft ionisation techniques such as electrospray ionisation (ESI)^6^ and matrix-assisted laser desorption/ionisation (MALDI)^7,8^ developed in the late 1980s. By the year 2000, these innovations, ESI-MS in particular, were used to analyse biomolecules with molecular weights up to 1 MDa. Today, nanoESI^9,10^ – in which the flow rate and droplet size are drastically reduced, resulting in far less sample consumption – as well as many other ionisation techniques, are coupled to many different MS instrument platforms for a broad range of applications.^5,11^

### 1.1 Top-down MS (TDMS) as a powerful tool for comprehensive protein characterisation

In the conventional bottom-up protein analysis approach, proteins are extracted, chemically or enzymatically digested, separated by liquid chromatography (LC), ionised *via* ESI, and analysed by MS, allowing identification, quantification, and PTM characterisation for many thousands of proteins. Information regarding protein isoforms, PTM stoichiometry, and combinatorial PTMs is, however, lost when using peptides as protein surrogates.^12,13^ The bottom-up approach has also been applied for higher-order structural characterisation using methods such as limited proteolysis,^14^ chemical crosslinking,^15^ and protein footprinting.^16^

‘Top-down’ mass spectrometry, which forgoes the digestion step, has proven to be the premier MS-based technology for unambiguous proteoform characterisation, enabling in-depth sequencing, the discovery of novel proteoforms, and quantification of disease-associated PTMs.^13^ While some technical challenges remain, developments in top-down protein analysis over the past few years have progressed its capability to unambiguously identify, characterise, and quantify thousands of proteoforms with high throughput. Recent developments in the growth, development, and applications in biomedical research of top-down protein MS are covered in several recent reviews.^5,12,13,17^ Simultaneously, another MS-based technology that has enabled key new biological insights is known as native MS. Native MS aims to preserve the solution structure of proteins and protein complexes during the transfer to the gas phase. Some of the key background terminology, abbreviations, and techniques relevant to the rapidly evolving fields of native and top-down MS are listed in Table 1.

**Table 1:** Brief explanation of some key terminology and techniques used in top-down and native mass spectrometry

Term	Meaning
TDMS	Top-down mass spectrometry; tandem MS of intact protein ions, with no enzymatic or chemical digestion step
TDP	Top-down proteomics; large-scale application of TDMS to (potentially) all proteins present in a cell, tissue, or organism, usually with the goal of understanding biological processes and gene expression control
CID/CAD	Collision-induced/collisionally activated dissociation; increasing the internal energy of ions by collisions with inert background gas molecules, a process in which energy is converted from translational to vibrational modes, resulting in dissociation of noncovalent and/or covalent bonds
HCD	Higher-energy collisional dissociation; used in Orbitrap instruments to distinguish ‘beam-type’ collisional activation in non-trapping multipoles from activation by resonant excitation in ion traps. Both processes involve collisions with background gas and generate qualitatively similar spectra. Although direct comparison of energy parameters is not trivial due to different instrument designs, HCD generally accesses higher-energy fragmentation pathways
ECD	Electron capture dissociation; fragmentation method for cations, based on gas-phase radical chemistry, in which a hydrogen-rich radical is formed by capture of (typically) a single low-energy (1–3 eV) electron by a biomolecular (typically a protein or peptide) cation
ETD	Electron transfer dissociation; similar to ECD, but the electron originates from a radical anion rather than an electron beam from a cathode emitter
EID	Electron ionisation dissociation; excitation of cations by fast electrons with energy at least 10 eV higher than the ionisation threshold of the cations
ExD	A general term referring to electron based activation, including ECD, ETD, and EID
UVPD	Ultraviolet photodissociation; method in which fragmentation is initiated by capture of (typically) a single ultraviolet (10–400 nm) photon. The exact mechanism depends on the photon wavelength, as described in the main text
IRMPD	Infrared multiphoton photodissociation; method in which fragmentation is initiated by capture of many infrared (780 nm to 1 mm; typically *ca.* 10 μm is used in practice) photons, leading to a gradual increase in internal energy and similar fragmentation behaviour to CID
SID	Surface-induced dissociation; method for ion activation/fragmentation based on accelerating an ion and colliding it with a surface within the mass spectrometer
Native MS	Native mass spectrometry; analysis by MS of biomolecules (primarily proteins) from non-denaturing solutions and using low-energy conditions in the source of the mass spectrometer, with the aim of preserving the higher-order structure in the gas phase
Native TD	Native top-down; gas-phase fragmentation of covalent bonds in an intact biomolecule or complex in a conformation-sensitive manner, so that information about higher-order structure can be inferred from the fragmentation pattern
nECD/nETD	Native electron capture/transfer dissociation; use of these two electron-based fragmentation methods for native TD mass spectrometry
Complex-up MS	The process of using ion activation to eject one or more monomers or ligands from a biomolecular complex without inducing significant cleavage of covalent bonds, so that, depending on the activation method used, monomer/ligand mass and/or subunit connectivity can be determined from the ejected species
Complex-down MS	The process of using ion activation to eject a monomer or ligand from a biomolecular complex, while inducing significant cleavage of covalent bonds (either in a single step with ejection or in separate stages), so that sequence or structural information on the ejected species can be obtained

### 1.2 Native mass spectrometry of protein complexes

For nearly thirty years, ongoing efforts have endeavoured to use MS to understand noncovalent protein complexes by transferring them into the gas phase without loss of higher-order structure.^5,18^ At its most basic level, native MS can return information on the makeup of protein complexes by providing molecular weights more accurately than conventional biophysical methods (*e.g.*, size-exclusion chromatography (SEC) or analytical ultracentrifugation) especially for heterogeneous samples. In relatively simple cases, this can suffice to indicate the number of monomers in a complex and determine differences in complex makeup.^19–22^ In addition to mass alone, the 3D shape of proteins and complexes can be simultaneously investigated by ion mobility-mass spectrometry (IM-MS).^23^ Previous studies have shown that IM-MS correlates with known protein structure,^24^ and that binding of ligands, cofactors, or metal ions can affect the observed structure, with important implications for *e.g.*, drug discovery.^25^ In addition, collision-induced unfolding (CIU), *i.e.*, increasing the internal energy of an ion prior to IM-MS analysis, allows concomitant study of conformational stability, providing more details than IM alone.^26^ While it is now commonly accepted that the stoichiometry of noncovalent protein complexes can be determined using native MS, the extent to which protein solution folding is retained, *i.e.*, the degree of ‘nativeness’ of the gas-phase ions, is more controversial. Ion mobility experiments show that the overall 3D shape of proteins – especially in the lower charge states naturally generated by ESI from non-denaturing solutions – is usually consistent with structures obtained from X-ray diffraction (XRD) and nuclear magnetic resonance (NMR) (although exceptions exist).^24^ Additional compelling evidence has been provided by ‘soft-landing’ experiments, in which gas-phase ions of large protein complexes are mass-selected and then gently decelerated and collected on a grid. Subsequent electron microscopy (EM) imaging then demonstrated preservation of native-like structures throughout the process of ionisation, dehydration, and soft-landing.^27,28^

Other analyses have further indicated that the overall structure of proteins is generally conserved in the gas phase. Using electron capture dissociation (ECD), McLafferty and co-workers have argued for refolding of small proteins in the gas phase to a non-native secondary and tertiary structure.^29^ Conversely, using electron transfer dissociation (ETD), Vachet and co-workers found that the gas-phase salt bridge pattern of small proteins was more consistent with the pattern present in the known native structure than any non-native alternatives,^30,31^ and gas-phase infrared spectroscopy carried out by von Helden and co-workers showed results consistent with preservation of alpha-helices and beta-sheets in native MS of myoglobin and beta-lactoglobulin, respectively.^32^ All of this supports the idea that, while significant expertise and care are needed, gas-phase structure may in fact reflect important aspects of native solution structure.

As ions formed under non-denaturing ESI conditions typically have low charge states, native MS instruments must be able to transmit and detect high-*m*/*z* ions. Today, this is possible using commercially available time-of-flight,^33^ Orbitrap,^34^ and Fourier transform ion cyclotron resonance (FTICR)^35^ instruments. These instruments can transmit large protein complexes without providing excessive activation that could compromise protein complex structure. In recent years, important progress in native purification methods such as native gel-eluted liquid fraction entrapment electrophoresis (GeLFrEE) separation,^36^ native gel electrophoresis,^37^ ion exchange chromatography (IEX),^38^ hydrophobic interaction chromatography (HIC),^39,40^ and online buffer exchange, has made these methods more applicable for complex mixtures and for characterisation of endogenous ligands.^36,41^

By using appropriate sample preparation, ionisation conditions, and instrumentation, many challenging analytes such as membrane proteins,^18,42^ intrinsically disordered proteins,^43^ highly dynamic or heterogeneous complexes,^19^ or very large systems such as intact virus capsids^44^ can all be investigated using native MS, as well as their associated proteoforms.^45^ Many of these are highly challenging to study by other analytical techniques such as NMR or XRD, and therefore the insights from MS can be vital to understanding these proteins and complexes. It is worth noting here that, while lower-resolution, MS-based methods are not necessarily less native than conventional methods – the crystalline state is far removed indeed from the native protein environment. These classical methods are also more prone than MS to sampling a single low-energy state or an ensemble average, and a combination of biophysical approaches can be needed to capture the dynamic nature of protein conformation. One aspect of protein structure to which native MS can be expected to be extended in the coming years is the study of protein quinary structure, which is defined by specific interactions in the crowded cellular environment that are weaker and more transient than those responsible for quaternary structure, and has recently been successfully investigated with non-native MS methods such as chemical crosslinking.^46,47^ Continued advances in sample processing and instrument development are expected to make these new experiments more routine for protein complex analysis in the near future. As will be discussed in the rest of this review, native ionisation has in recent years been increasingly combined with top-down protein fragmentation, allowing probing of different structural levels and relating sequence information to higher-order structure and complex formation.

## 2. Gas-phase activation of intact, native proteins and complexes

### 2.1 Activation of protein complexes without backbone cleavage for quaternary structure (‘complex-up’ methodology)

#### 2.1.1 Native MS alone provides limited information for heterogeneous complexes

The complex-up strategy aims at subunit dissociation of noncovalent complexes without cleaving covalent bonds.^17^ Native MS without breaking up the complexes only provides limited information regarding quaternary structure and is largely blind to subunit connectivity and location of ligand binding within the complexes. For unknown complexes, intact mass alone is not enough for determining stoichiometry and composition. In addition, gentle tuning conditions used to maintain structural integrity of noncovalent complexes can result in insufficient desolvation, peak broadening, and increased uncertainty in mass determination. For fragile complexes, the result of this is that the achievable mass resolution may be too low for precisely defining the binding of small ligands. Recent publications by the Kelleher^48^ and Heck^49^ laboratories have demonstrated the use of charge detection MS of noncovalent complexes on Orbitrap instruments. In these experiments, small numbers of ions were allowed in the trap, allowing highly repeatable mass measurement of individual ions. A histogram of the single-particle centroid masses constructed after thousands of these measurements provides significantly (approximately an order of magnitude) higher resolving power than conventional Orbitrap MS. Still, heterocomplexes that have multiple subunits with very similar masses are difficult to characterise just from the intact mass, especially when there is high uncertainty in mass measurement.^50^ Another limitation of mass measurement alone of intact proteins and complexes is that their (average) masses might shift slightly due to natural variations in isotopic abundance, an effect which is able to cause mass shifts greater than the accuracy of modern high-end mass spectrometers.^51^

Solution disruption *via* addition of chemical denaturants has been used to partially dissociate native protein complexes into subcomplexes, including successful applications to RNA polymerase and exosomes.^52,53^ This technique enables a simple way to access the subunit connectivity without changing the downstream native MS detection method. Because the dissociation occurs in solution, this method may not be easily applicable to highly heterogenous samples, as released subcomplexes cannot be tracked to their originating precursors. Furthermore, the protocol for partial denaturation requires optimisation for each complex and can fail for proteins that are resistant to mild denaturants or precipitate easily upon denaturation. Other solution-phase methods exist to study higher-order protein structure by subsequent MS analysis, for example chemical crosslinking,^54^ protein footprinting methods including fast photochemical oxidation of proteins (FPOP),^16^ and hydrogen–deuterium exchange;^55–58^ however, these are beyond the scope of this perspective. Integration of information from different native and non-native techniques can provide valuable structural insights.^59,60^

#### 2.1.2 Collisional activation induces protein unfolding and subunit release

The term ‘complex-up’ was coined in 2019;^17^ however, early examples of subunit release *via* activation of protein complexes in the gas phase were reported in 1994 by Smith and co-workers.^61^ Under harsh source conditions, several model tetrameric complexes were dissociated into monomers and trimers. This was surprising because the trimers were not known to be physiologically relevant. This dissociation pattern of monomer stripping appeared to be ubiquitous in several early studies using gas collision to activate the complexes (known as collision-induced dissociation, CID, collisionally activated dissociation (CAD), or higher-energy collisional dissociation, HCD in some instruments), and was also seen for blackbody infrared radiative dissociation (BIRD).^62^ Essentially, a monomer in the complex was stripped from the complex, leaving behind the stripped (*n* − 1)-mer (*n* is the number of subunits in the precursor complex).^61,63,64^ The stripped monomers carry away a disproportionate fraction of the total charge relative to their mass. This seemingly odd pattern was described as ‘asymmetric’ dissociation and was studied in detail by several follow-up reports.^62,65^ Accumulating experimental and computational studies have suggested that charge plays an important role in gas-phase protein unfolding and dissociation.^62,65–67^ The mechanism of charge migration to the ejected monomer is not fully understood, but mobile charge^62,68,69^ and salt bridge rearrangement theories^70,71^ have been proposed.

CID has been used to release subunits from protein complexes for confirming complex composition. For example, the ubiquitous monomer-stripping pattern was used to activate αB-crystallin complexes with polydisperse stoichiometry (primarily 24–33 mer).^72^ As larger oligomers carry more charge in native MS, the signals for all these oligomers end up as an overlapping, unresolvable cluster around *m*/*z* 10 000. The released (*n* − 1)-mers, (*n* − 2)-mers, and (*n* − 3)-mers from sequential monomer stripping could, however, be mass resolved and from these, the stoichiometry of the intact complexes was inferred.^72^ Although CID can be used to identify the composition of unknown complexes, the dissociation may be incomplete and insufficient to release all subunits of multimeric hetero-complexes.^50,73^ Typically, subunits at the periphery are preferentially ejected in CID.^74–76^ Because of the significant unfolding and the ubiquitous monomer stripping dissociation pattern, extracting subunit connectivity and architecture from CID data is usually not straightforward.

#### 2.1.3 Surface-induced dissociation reveals subunit connectivity and ligand binding

**Fig. 1:**
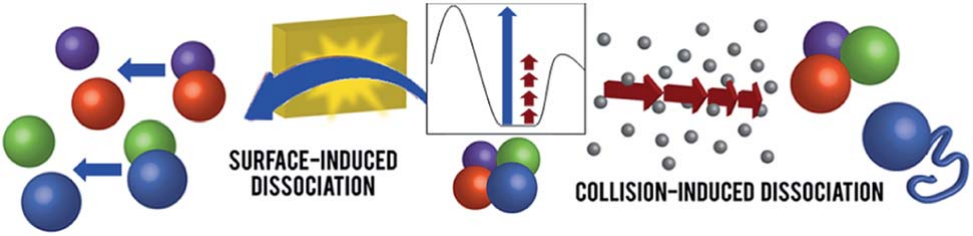
Schematic representations of CID and SID of noncovalent protein complexes. A hypothetical potential energy diagram is shown in the inset (reaction coordinate on *x* axis, potential energy on *y* axis, arbitrary energy scale). In CID (on the right), protein complexes undergo many steps of collisions, resulting in structural rearrangement, unfolding, and monomer ejection. Rapid activation in SID (left) allows direct dissociation into folded subunits. Adapted with permission from ref. 90. Copyright 2018 American Chemical Society.

Surface-induced dissociation (SID), in which proteins collide with a surface target, can produce folded subunits with minimal structural rearrangement for a number of model protein complexes.^77^ SID is more efficient than CID in converting kinetic energy of an ion to internal energy because of the larger mass of a surface target compared to neutral gas molecules. Refractory protein complexes (typically those with strong charge–charge interactions including protein–RNA/DNA complexes) are difficult to dissect by CID but can be dissociated by SID.^78,79^ In addition, the activation in SID occurs on a much shorter time scale than (low-energy) CID, in which protein ions undergo many lower-energy collisions (Fig. 1). This rapid activation in SID allows protein complexes to be dissected into subcomplexes prior to significant structural rearrangement.^77,80,81^ The released subcomplexes therefore provide information on the connectivity of subunits in the precursor.^82,83^ For example, the streptavidin tetramer preferentially dissociates into dimers in SID, which is representative of the native ‘dimer-of-dimers’ structure of the complex. SID has been shown to be helpful for mapping the topology of designed heterocomplexes,^84,85^ dissecting the assembly mechanism of transthyretin,^86^ and the structural characterisation of 20S proteasome orthologue complexes from different species.^22^ SID was also used to localise noncovalent ligands in multimeric complexes.^50,87,88^ However, care must be taken to minimize structural rearrangement before SID. Harsh conditions in the source or transfer optics for achieving the best mass resolution could over-activate the complexes and change their shape significantly, which is measurable by ion mobility. Over-activated complexes will generate different SID spectra from their original structures.^89^

Systematic examination of model complexes showed that the SID collision energy required to cleave a given interface is correlated with the interface strength calculated from the (known) structure of the complex.^82^ Weaker interfaces will therefore be cleaved at lower collision energy than stronger interfaces in SID. The structurally informative dissociation by SID enables quaternary structure characterisation of unknown proteins that are recalcitrant to classical structural biology techniques. Toyocamycin nitrile hydratase (TNH) and bacterial biominerialisation enzyme Mnx are two heterocomplexes that resist crystallization. Their mass (86 kDa and 210 kDa, respectively) also puts these complexes in a range that is too large to be easily studied by NMR, but too small for cryo-EM. SID experiments of these complexes were quick, with data acquisition typically on the order of minutes to hours, and provided critical information to define their quaternary structures.^50,73,88,91^

**Fig. 2:**
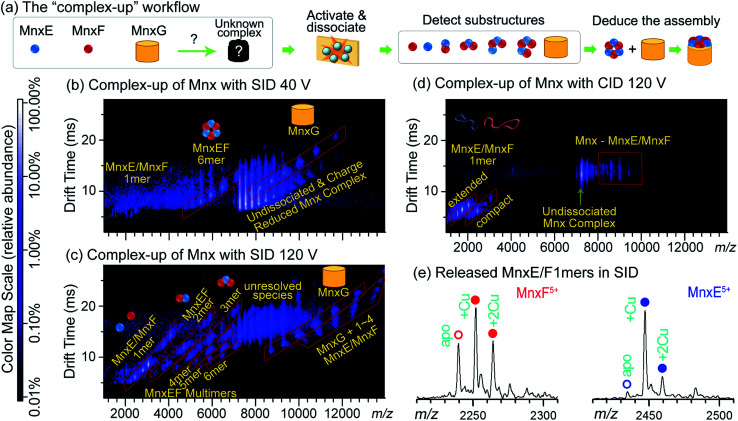
(a) ‘Complex-up’ workflow for determining quaternary structure of Mnx. Gas-phase activation of the complex resulted in dissociation into substructures, knowledge of which can reveal how the complex is assembled. (b) SID of Mnx with collision voltage of 40 V. The spectrum has the *m*/*z* on the horizontal axis and the drift time measurement (related to size-to-charge ratio) from ion mobility separation on the vertical axis. Major assigned species are highlighted in red parallelograms and noted with cartoon structures. Other species are noted with yellow text. The same format is used for (c and d). At relatively low SID collision voltage, Mnx was dissected into MnxE_3_F_3_ and MnxG. (c) SID spectrum of Mnx with 120 V collision voltage. Under these conditions, MnxE_3_F_3_ dissociated into smaller substructures, mostly heterocomplexes. (d) CID spectrum of Mnx with 120 V collision voltage, showing exclusively monomer stripping. Most of the monomers show extended (*i.e.*, unfolded) conformations. (e) Extracted mass spectra for the released MnxE/F monomers from (c). The number of bound Cu atoms can be easily determined, and differs between MnxE and MnxF. Figure adapted with permission from ref. 50.

Fig. 2a illustrates how the data from complex-up MS were recently used to study the previously uncharacterised heterocomplex Mnx, which consists of three proteins: MnxE (12.2 kDa), MnxF (11.2 kDa), and MnxG (138 kDa). MnxG is homologous to multicopper oxidase, a monomeric enzyme. MnxE and MnxF have no known homologues or functions, but are essential for the stability of the Mnx complex. Because of the similar mass of MnxE and MnxF, the stoichiometry could not be confidently assigned from size-exclusion chromatography, or – due to the peak-broadening effects discussed previously – even native MS alone. After mass-isolation of the Mnx complex, SID dissected Mnx into MnxE_3_F_3_ hexamer and MnxG at low collision energy (Fig. 2b), suggesting the complex stoichiometry to be MnxE_3_F_3_G. With increased collision energy in SID, MnxE_3_F_3_ further dissociated into subcomplexes following a similar pattern to other symmetric ring complexes (Fig. 2c). The SID data were used to map the subunit connectivity and architecture, allowing a structural model to be built (Fig. 2a). The resolution of the model can be improved to an all-atom level *via* computational tools, constrained by additional experimental data such as collisional cross section from ion mobility and solvent exposed surface area determined by footprinting techniques as described in Section 2.1.1. In contrast, CID of Mnx showed almost exclusively monomer stripping (MnxE and MnxF), providing limited information for mapping the assembly (Fig. 2d). Notably, MnxE and MnxF monomers released from Mnx by SID showed different Cu-binding stoichiometry (Fig. 2e). MnxE strongly binds to one Cu mostly, while MnxF can weakly bind multiple Cu atoms. The different binding behaviour between MnxE and MnxF revealed by complex-up experiments suggest that the two unknown proteins presumably have different functions. Unlike SID, CID experiments were not able to faithfully capture the metal-binding properties of MnxF, as, during monomer unfolding, the weakly bound Cu in this subunit was lost.^50,88^

Recently, SID was also applied to characterise the subunit arrangement of a plant pseudoenzyme-enzyme hetero-complex between PDX1.2 and PDX1.3 in *Arabidopsis*.^92^ The pseudoenzyme PDX1.2 lost its activity due to mutation of a few key residues at the active site but is nearly identical structurally to the active enzyme PDX1.3. The two proteins form hetero-dodecamers with varying stoichiometry. Both XRD and cryo-EM suffered from the statistical disorder and were not able to distinguish the two types of subunits in the hetero-complexes because of their highly similar shapes and the heterogeneity in stoichiometry.^92^ However, their different masses can be readily differentiated by MS. SID of the isolated hetero-dodecamers also revealed the symmetry of the subunits within the complex and shed light on the mechanism of the hetero-association. All these examples show that SID is effective for quaternary structure study following the ‘complex-up’ strategy. Such experiments complement other structural biology techniques, especially for heterogenous complexes that are difficult to resolve by any single technique. So far, one major factor that has limited the use of SID in practice is the more limited availability of this technique compared to other ion activation methods, although recent work by Wysocki and co-workers has simplified the design and operation of SID.^93^ The new design was successfully incorporated into several commonly used instrument models for native MS. The first commercially available SID-enabled instrument was recently announced (SELECT SERIES Cyclic IMS; Waters Corporation, Milford, MA, USA) and others are expected to follow in the near future.

#### 2.1.4 Other activation methods and important factors for complex-up MS

Other than CID and SID, photo-activation by ultraviolet (UVPD) and infrared multiphoton photodissociation (IRMPD) have also been used in complex-up experiments. While exceptions have been reported,^94^ electron-based activation generally does not cause significant disruption of higher-order structure^35^ and is thus ineffective for complex-up. Intramolecular energy redistribution after conversion of photon energy to vibrational modes is thought to be responsible for breaking of noncovalent interactions in UVPD.^95^ IRMPD of protein complexes, on the other hand, has produced similar results to CID, likely because of the low energy of infrared photons, of which dozens or even hundreds are absorbed to cause dissociation.^96^ In contrast, 193 nm UVPD of several model protein complexes showed CID-like asymmetric dissociation at low pulse energy, but changed to more symmetric dissociation (SID-like) at higher pulse energy.^97,98^ This change of dissociation behaviour as a function of input energy in UVPD is reminiscent of the mechanistic difference between the slow heating in CID and rapid heating in SID. Even though SID has been shown to induce dissociation of complexes with minimal unfolding, the monomer stripping pathway indicative of unfolding can also be observed in SID, especially for large protein complexes. Previously, a ‘shattering’ mechanism (*i.e.*, prompt fragmentation/dissociation distinct from slow collisional and thermal activation) was proposed for SID of peptides,^99^ but dissociation may occur at a much slower rate after activation for larger molecules with high degrees of freedom and significant intramolecular energy redistribution/relaxation. SID of large protein complexes could also result in multiple, inelastic collisions like those seen for cluster ions.^100^ The energy deposition could be affected depending on how the protein ions interact with the surface.^101^

In addition to the differences in activation techniques, the charge of protein complexes is another important factor in complex-up experiments.^66,67,98,102^ Charge reduction through solution additives and gas-phase reactions can supress protein unfolding, and generally help SID by increasing the percentage of structurally informative products.^66^ In contrast, subunit dissociation is suppressed in CID for charge-reduced precursors. Instead, CID tends to benefit from supercharging, allowing more SID-like dissociation.^67^ Computational and theoretical studies have indicated that charge can move upon activation, as further discussed in Section 2.3. The energy landscape of protein complexes in the gas phase is thus strongly affected by charge, resulting in different behaviours with different activation methods. When the goal is to study quaternary structure with a complex-up strategy, conditions should therefore be optimised to minimize unfolding and structural rearrangement.

### 2.2 Sequencing of ejected proteins from native complexes (‘complex-down’ approach)

#### 2.2.1 MS^*n*^ for sequence and stoichiometry of native protein complexes

While mass measurement of monomers and noncovalent (sub)complexes is informative, oftentimes complementary sequence analysis is desired to identify the component proteoforms. There are multiple ways to obtain this information from intact proteins. Top-down MS under denaturing conditions yields important information on sequence and PTMs, but information regarding noncovalent interactions or protein folding is lost. Efficient sequencing can be combined with obtaining information on noncovalent complex stoichiometry through MS^*n*^ (*n* = 3 or more) analysis of complexes ionised under non-denaturing conditions. This type of experiment has been referred to as ‘complex-down’^17^ MS or recently as ‘nativeomics’.^103^ The extra layer of information afforded by fragmentation of covalent bonds compared to the methods described in Section 2.1 can facilitate a greater understanding of protein complex function.

**Fig. 3:**
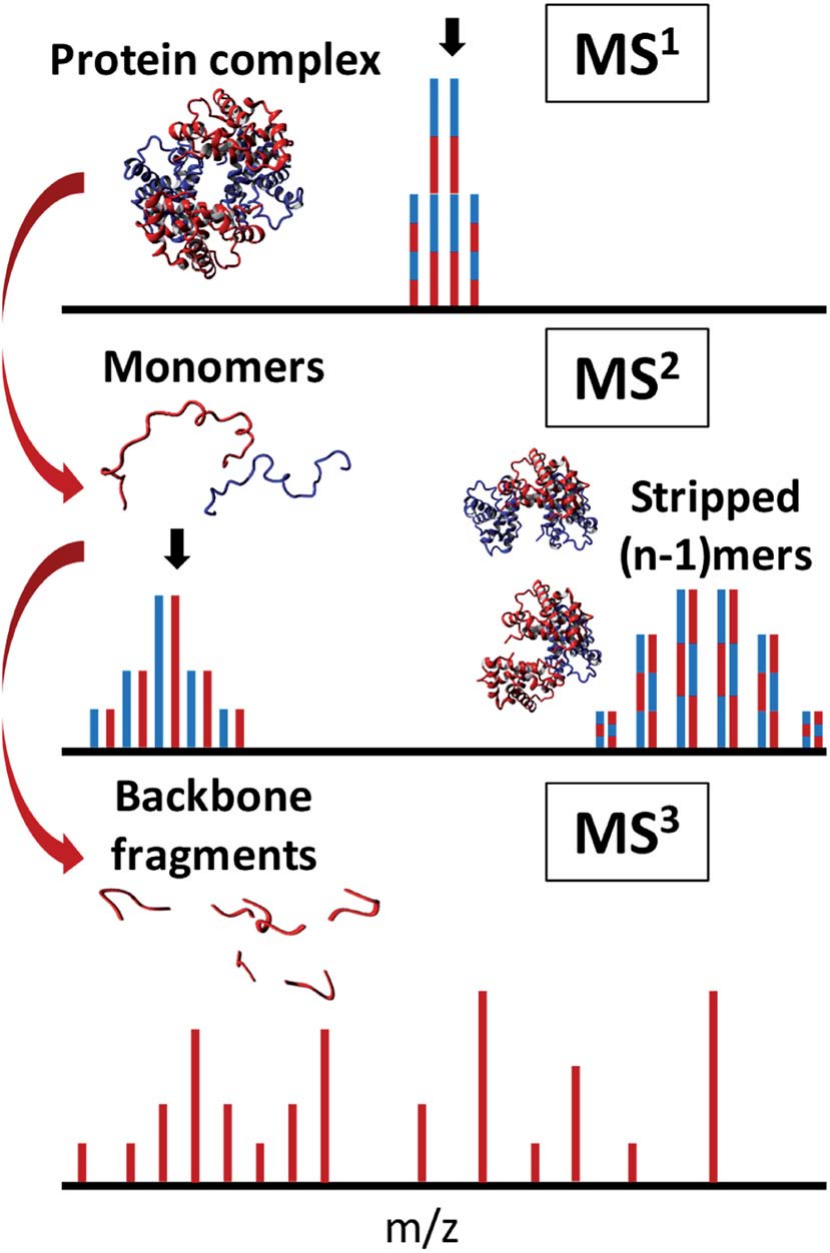
Principle of complex-down MS. After native ESI of a protein complex and isolation (indicated by black arrow) of a specific charge state (top), monomers are ejected and again isolated (middle), enabling efficient sequencing (bottom).

The first step of these experiments is to transfer a noncovalent complex from the solution phase to the gas phase without excessive disruption of its higher-order structure.^103^ Next, the internal (vibrational) energy of the complex is increased sufficiently to induce ejection of monomers or non-covalent ligands without breaking covalent bonds. Normally, activation is provided by techniques such as CID or SID. CID can be implemented either after a specific precursor *m*/*z* is isolated in the gas phase, or through elevated acceleration voltage of all ions in the source. In the latter case, the technique is often referred to as in-source dissociation (ISD), which generally requires highly purified samples or online separation so that the products can be traced to the precursor unambiguously. As described in Section 2.1, the charge density of the ejected subunits is usually higher than that of the original complex, making them amenable to sequencing by several commonly-used ion activation methods. The ejected ion – monomer or ligand – is subsequently (re-)isolated and fragmented in a (pseudo-)MS^3^ or MS^4^ workflow, providing in-depth information on *e.g.*, phosphorylation sites,^104^ metal ion binding,^36^ or sequencing of peptide ligands (see Fig. 3).^105^ In 2013, Kelleher and co-workers performed complex-down analysis of the GroEL 14-mer (801 kDa).^106^ It was found that GroEL monomers could be ejected from the complex in the source and further fragmented with HCD. Sequence-informative fragments could be readily observed in the low-*m*/*z* region of the mass spectrum with this workflow. More recently, Heck and co-workers obtained sequence information on the *Aquifex aeolicus* lumazine synthase (AaLS) virus-like nanocontainer (>1 MDa) with UVPD fragmentation, resulting in a mix of monomer ejection and backbone fragmentation.^107^ It was found that optimal collisional cooling and trapping before UVPD fragmentation allowed for efficient detection of intact virus nanocontainers, monomers, and sequence fragments. Combining knowledge of the intact mass of protein complexes from native MS with accurate mass measurement of the ejected, desolvated, highly-charged monomers (MS^2^), and sequence information (MS^3^) in this manner, allows for the determination of the exact proteoform composition of the complex.

#### 2.2.2 Sequence elucidation and modification location analysis through complex-down MS

In addition to identifying the primary sequence of subunits, complex-down analysis can elucidate sequence abnormalities such as deletions and mutations in native protein complexes. These data can help characterise the structure and function of protein complexes. Sharon and co-workers found novel sequence information on the alpha subunit of the rat 20S proteasome with complex-down analysis.^22^ Notably, they found N-terminal acetylation and removal of the last two amino acids on the C-terminus, complementing data that was absent from cryo-EM, which did not have enough resolution to discern these features. In another example of how complex-down analysis has been shown to aid in the identification of protein complex mutations by providing deep sequence information on ejected monomers, Compton and co-workers demonstrated how complex-down analysis of proteins with regulatory post-translational modifications leads to more accurate structural and functional characterisation of those complexes.^36^ Sequence analysis of the triosephosphate isomerase complex indicated the presence of a proteoform that was not modified, a proteoform that was phosphorylated at serine 20, and a proteoform that was N-terminally acetylated. The phosphorylated and acetylated proteoforms did not dimerise with themselves or each other, indicating that phosphorylation at serine 20 and N-terminal acetylation act to inhibit the dimerisation of the complex.^36^

Sharon and co-workers have used complex-down analysis to determine how yeast cells use phosphorylation to regulate certain cellular pathways under different growth conditions.^104^ It was found that the level of phosphorylation of the fructose-1,6-bisphosphatase 1 (FBP1) complex differed depending on whether the cells were grown on carbon-starved media, glucose media, or were heat-shocked. Complex-down analysis of the monomers indicated that phosphorylation on Ser12 or Thr13 was highly expressed in cells that were grown on glucose media. Phosphorylation at Ser12 is known to deactivate this complex, indicating that the complex is deactivated under these conditions and the cells readily switch from performing gluconeogenesis to glycolysis. In all these examples, complex-down analysis efficiently located key modifications and/or sequence variants on native protein complexes, illuminating key structural and functional characteristics of those complexes.

#### 2.2.3 Complex-down for characterisation of membrane proteins

**Fig. 4:**
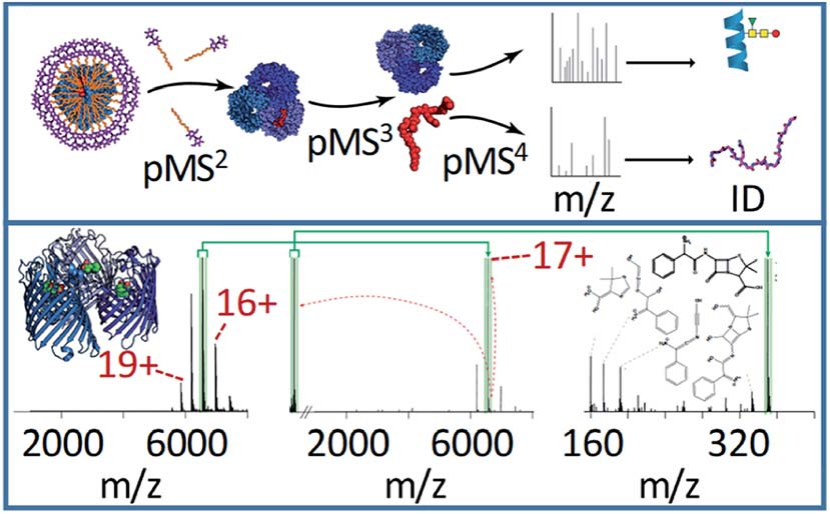
The general workflow for complex-down MS of membrane protein assemblies is shown in the top panel, including the in-source activation step (referred to by the authors as p(seudo-)MS^2^) needed to strip away detergent molecules. The bottom panel shows the application of this method to the 17+ charge state of ampicillin-bound OmpF. The pseudo-MS^3^ spectrum (middle) in this case shows the mass of the ligand, and the pseudo-MS^4^ (right) shows characteristic fragment ions, allowing confident ligand identification. Adapted with permission from ref. 103. Copyright 2020 Springer Nature.

Generally, complex-down analysis is performed on soluble protein complexes that are relatively easy to dissolve and spray in native MS buffers. Recently, this technique has been extended to membrane proteins by adding detergents or lipids in the solution to prevent protein precipitation and to preserve their native structure. Carefully tuned collisional activation is used to eject intact membrane protein complexes from detergent micelles so that the membrane protein can be efficiently analysed.^108^ Increasing the level of this activation leads to the ejection of protein monomers, allowing complex-down sequencing (see Fig. 4). Recently, Robinson and co-workers applied this workflow to show that MS^*n*^ analysis was able to identify a lipid molecule that was bound to the outer mitochondrial membrane translocator protein complex.^103^ In-source activation ejected the membrane protein from the detergent micelle encapsulating the protein; isolation and activation of the membrane protein complex *via* HCD ejected the lipid molecule, which was in turn isolated and subjected to HCD or CID, allowing it to be identified. The lipid was found to be a phosphatidylethanolamine 34:1, which fit well with the existing crystal structure. In standard omics experiments, proteins and lipids were extracted separately and characterised by LC-MS; however, using the complex-down strategy, the association between protein and lipid could be directly identified in their native context, providing insight into their biological role.

Brodbelt and co-workers used UVPD for effective sequencing of aquaporin Z monomers ejected from the native tetramer.^109^ The sequence coverage for monomers was increased by 21% in this way compared to direct native TD UVPD of the tetramer. This increase was presumably due to disrupted noncovalent interactions between subunits (see Section 2.3 for more on how these interactions affect the native TD fragmentation pattern). In some cases, complex-down analysis has been shown to map important regions of native proteins. Sobott and co-workers recently studied three membrane proteins, *i.e.*, the pentameric mechanosensitive ion channel of large conductance (MscL), the tetrameric Kirbac potassium channel, and the hexameric hepatitis C p7 viroporin. Performing CID-based complex-down MS, they found that *b* and *y* fragment ions mainly stemmed from dissociation in the membrane-spanning regions of the monomers.^110^ This was consistent with earlier work by Kelleher and co-workers, who performed top-down LC-MS of denatured integral membrane protein monomers, and found that transmembrane domains were more likely to fragment in collisional than electron-based dissociation.^111^

#### 2.2.4 High-throughput complex-down of heterogeneous protein samples

Recently, high-throughput native separation techniques including GELFrEE,^36^ HIC,^39,40^ SEC,^112^ capillary zone electrophoresis (CZE),^112^ and IEX^38^ have efficiently aided in the analysis of heterogeneous protein mixtures. Kelleher and co-workers demonstrated the use of native GELFrEE coupled to MS for the characterisation of protein complexes from cobra venom.^113^ Complex-down analysis enabled the characterisation of protein complexes, and in some cases allowed identification of glycosylated and metal-bound proteoforms. In a different example, Sun and co-workers showed that SEC and CZE can separate multiple proteins and protein complexes before analysis with complex-down mass spectrometry on a lysate of *E. coli*, resulting in the identification of 672 proteoforms and 23 protein complexes.^112^ These examples illustrate how, by preserving the native structure of complexes, these high-throughput techniques can yield critical information regarding complex formation and ligand binding that is not easily accessible by conventional proteomics techniques. New developments in MS instrumentation,^113^ spectrum deconvolution software,^112^ and fast peak identification software^113^ are expected to facilitate high-throughput complex-down studies becoming more routine in the future.

Data analysis has so far been a particular bottleneck in these approaches, as native MS spectra often do not have isotopic resolution, and so common software for small molecule and peptide analysis cannot be used to convert *m*/*z* to intact mass. Spectral interpretation of native MS has largely relied on manual analysis, although deconvolution software such as UniDec and iFAMS has been developed in recent years.^60,114^ Likewise, analysis of top-down MS data has largely involved manual analysis or manual validation due to the complexity of the data. Data interpretation for complex-down spectra is similar to TDMS of denatured proteins; therefore, many existing software packages for TDMS can be retrofitted for complex-down data, including ProSight PTM 2.0,^115^ ProSight Lite,^116^ MASH Suite,^117–119^ ProteinGoggle,^120^ TopPIC,^121^ Informed-Proteomics,^122^ Masstodon,^123^ and others. Even then, some unique challenges unique to complex-down remain, including the need for multi-stage activation, poorly resolved precursor complexes, and low intensity peaks, and so manual analysis or validation still plays an important role in practice. An up-to-date overview of software packages for analysis of top-down data can be found on the webpages of the Consortium for Top-Down Proteomics (https://www.topdownproteomics.org) and an excellent review was recently published.^12^

### 2.3 Conformation-sensitive fragmentation of native complexes for secondary and tertiary structure

#### 2.3.1 Electron- and photon-based activation can directly probe gas-phase higher-order structure

The methods discussed in Section 2.2 rely on gas-phase fragmentation of the protein backbone, but only after the higher-order structure of a protein complex has been largely annihilated. The ‘native top-down’ strategy also refers to fragmentation of the protein backbone, but fragmentation methods are directly applied to native proteins or complexes instead of unfolded proteins. Native TD generally results in less extensive fragmentation than denatured TD because (1) the lower charge states of native species in ESI have a negative impact on the efficiency of most fragmentation methods, and (2) the noncovalent interactions (salt bridges, hydrogen bonds, *etc.*) that characterise higher-order structure can protect parts of the protein from fragmentation and/or prevent the release of fragments. Due to the second factor, the lack of fragments in certain regions of the protein can inform on higher-order protein structure.^35^

Electron-based dissociation (ExD) is useful for probing protein structure because it generates backbone fragments without annihilating the higher-order structure of the protein. In ECD, protein ions capture low energy (1–3 eV) electrons, leading to the formation of *c/z*˙ fragment ions.^124^ Electron ionisation dissociation (EID) is an electron-based fragmentation method that uses higher-energy electrons to fragment proteins generating *a*/*x* and *b*/*y* ions in addition to *c/z*˙ ions.^125^ EID offers more extensive fragmentation than ECD and, as electron energy can be tuned through user-accessible instrument parameters, both experiments can be performed using the same instrumentation. In addition, it was recently reported that EID of proteins results in more internal fragments than ECD which could help with protein sequence coverage.^126^ ETD is mechanistically similar to ECD, but uses a radical anion rather than a cathode to provide electrons.^127^ As in ECD, the transfer of the low-energy electron typically results in *c/z*˙ fragment ions.

Native TD can be performed using UVPD, and light sources – usually lasers – with different wavelengths have been reported for protein characterisation. Most of the reported native TD work has used 193 nm UVPD (ArF excimer laser), which yields *a*/*b*/*c*/*x*/*y*/*z* ions from the mixed mode of both vibrational and electronic excitation.^95^ In comparison, UVPD with 157 nm photons (F_2_ excimer laser) appears to be more effective at generating *a*/*x* ions and has specificity towards disulphide bond cleavage.^128,129^ UVPD at wavelengths of 213 nm ^130^ and 266 nm ^131^ has also been reported for native TD of small proteins. In the following paragraphs, we will consider how different aspects of higher-order structure can be probed using native TD fragmentation.

#### 2.3.2 Native TDMS for secondary and tertiary structure

Fourier transform ion cyclotron resonance instruments can readily be used to perform ECD because low-energy electrons can be efficiently trapped by the static electromagnetic field in the ICR cell. Early experiments used ECD to probe structural changes in ubiquitin^29^ and cytochrome *c*^132^ after being transferred into the gas phase. It was found that these proteins show more fragmentation with an increase in source temperature and pre-ECD infrared activation. This indicated that these proteins start to unfold in the gas phase when their internal energy is increased by these ‘slow’ activation methods. These experiments on small monomeric proteins clearly demonstrated the feasibility of accessing secondary and tertiary structures using MS techniques and spurred research on larger proteins and protein complexes.

**Fig. 5:**
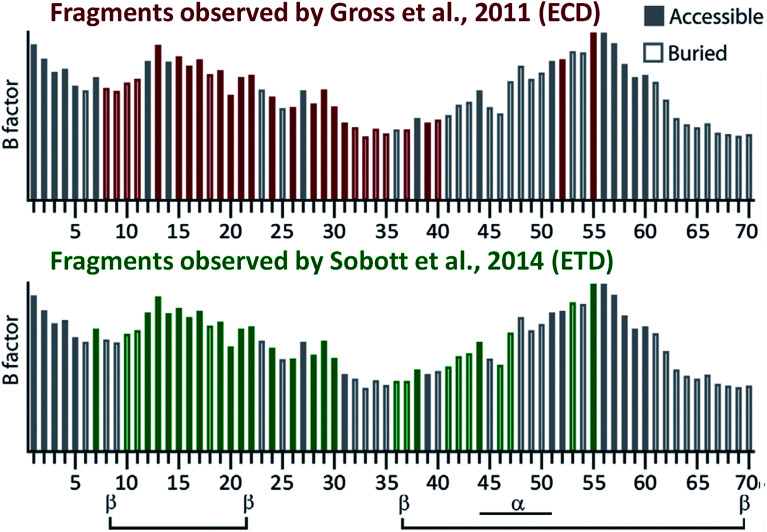
Comparison of the native TD fragmentation pattern observed in native ECD^136^ and ETD^137^ of the ADH tetramer and correlation to surface accessibility (hollow or filled bars) and local backbone flexibility (*B* factor; height of the bars) calculated from the crystal structure of the complex (only the first 70 N-terminal residues are shown, as this is where nearly all fragments originated from). Secondary structure elements from the crystal structure are shown at the bottom of the figure. Adapted with permission from ref. 138. Copyright 2018 John Wiley and Sons.

ExD techniques have been used to probe the secondary and tertiary structure of larger proteins and protein complexes in the gas phase. It has been reported that even though ExD techniques may fragment the backbone of the protein, interactions such as salt bridges^30^ and disulphide bonds^133^ can hold fragments together, preventing their release and detection in MS. This phenomenon is known as ‘electron capture/transfer with no dissociation’ (ECnoD or ETnoD, respectively). This principle can be used to probe secondary and tertiary structure. Barran and co-workers have shown that the stability of certain secondary structure elements of proteins can be probed by fragmenting different charges states.^134^ It has also been found that the location of disulphide bonds can be mapped with ExD fragmentation.^133,135^ ExD techniques have also been shown to probe tertiary structure on protein complexes such as alcohol dehydrogenase (ADH). ECD^136^ and ETD^137^ of native tetrameric yeast ADH both yielded primarily N-terminal fragments, while virtually no fragmentation in the C-terminal region was observed. For the C-terminus, this behaviour can be easily rationalised, as it is buried in the interior of the complex. To rationalise relative fragment abundances from the N-terminal region, explanations have been proposed based on local backbone flexibility and surface exposure (see Fig. 5).^136–138^ ExD fragmentation has also been utilized to probe the structure of other protein complexes such as haemoglobin^139^ and glutamate dehydrogenase.^35^ These examples clearly show that ExD can provide useful structural information about proteins and complexes.

Similar to electron-based activation, 193 nm UVPD has been shown to cleave preferentially at flexible and exposed regions of proteins and protein complexes. However, 193 nm UVPD generally shows higher sequence coverage than ExD and can yield fragments even in more protected regions of proteins.^125,140,141^ The component of vibrational excitation in 193 nm likely improved the release of fragments due to more efficient disruption of noncovalent interactions than in ExD. Nonetheless, high-intensity fragments are generally correlated with surface accessibility in UVPD, and shifts in fragment intensities indicate conformational changes.^141–143^ By quantifying the changes in fragmentation efficiency, subtle structural effects of metal-/ligand-binding can be probed with 193 nm UVPD.^144,145^

**Scheme 1:**
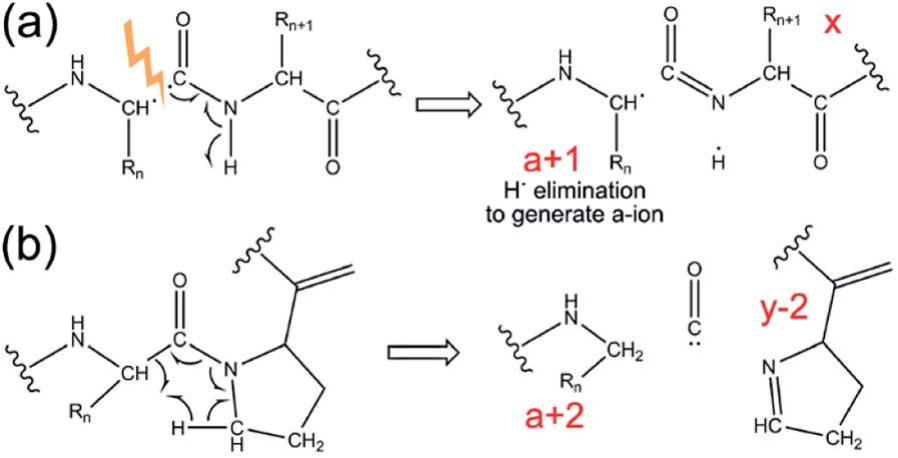
Mechanism for generation of (a) *a* + 1 ion, and (b) *a* + 2 ion in UVPD. Adapted with permission from ref. 141. Copyright 2020 American Chemical Society.

A unique feature of 193 nm UVPD is that the *a*-type fragment ions are sensitive to protein secondary structure. Previous studies on proline-containing peptides showed that *a* + 1 and *a* + 2 ions (*i.e.*, *a*-type ions carrying one or two additional hydrogen masses, respectively, relative to their canonical structure) can be detected (see Scheme 1). Upon homolytic cleavage in UVPD, odd-electron *a* + 1 ions are produced. These are thermodynamically unstable and can eliminate a hydrogen atom to form the commonly detected *a* ions. Amino acid structures and secondary structures both affect the lifetime and the detectability of *a* + 1 ions.^146–148^ The rigid backbone of proline was believed to be responsible for the *a* + 2 ions, which are formed as the alpha-carbon abstracts a hydrogen atom from a nearby residue.^148–150^ These behaviours have recently been shown to translate from the early peptide studies to model protein complexes.^141,146^ The increased relative abundance of *a* + 1 to *a* ions was correlated with hydrogen bonding motifs in small monomeric proteins.^146^ Turn structure in proteins was also suggested to be responsible for formation of *a* + 2 ions in the absence of proline.^141^ Therefore, it is possible to extract such spectral features from UVPD experiments to obtain secondary structural details of protein complexes in the gas phase.

#### 2.3.3 Ligand binding sites and subunit binding interfaces through native TD

Both ExD and UVPD can identify ligand binding sites in native TD through monitoring backbone fragments that retain the noncovalent ligand. Since these ligands are generally weakly bound to proteins and protein complexes, activation through CID tends to disrupt the ligand binding, causing information about the binding site to be lost. In contrast, ExD and UVPD can dissociate the peptide backbone while preserving the ligand. In the fragmentation spectrum, the larger *apo* fragments (*i.e.*, long sections of the protein sequence without ligand) and the smaller *holo* fragments (short stretches with the ligand) can together define the site (or region) of the bound ligand.^136,140,151^ Using this analysis, it is possible to localise binding sites on proteins and complexes, providing insight into the function of ligands.

Loo and co-workers demonstrated for the first time in 2006 that ECD could be used to localise noncovalently bound spermine on the amyloidogenic protein α-synuclein.^151^ Soon after that, Sadler and co-workers showed that binding sites of the drug cisplatin can be localised on peptides with ETD fragmentation.^152^ Since then, ECD has been used to pinpoint NAD^+^ on alcohol dehydrogenase^153^ and small aggregation-inhibiting compounds on amyloid proteins.^154,155^ Correlation of these binding sites with structural changes can suggest possible mechanisms of noncovalent ligand binding.^155^ Several of these examples also illustrate the capability of native MS-based methods to probe intrinsically disordered proteins and their complexes. More recently, UVPD has been used to localise noncovalent ligands on protein complexes, such as haem on myoglobin,^143^ NADPH and methotrexate on dihydrofolate reductase,^142^ and GTP on eIF4E.^140^ These examples clearly show that ExD and UVPD preserve noncovalent ligands when cleaving the peptide backbone and can readily localise these ligands on proteins and protein complexes.

Fragmenting protein/metal ion complexes can aid in the localisation of metal ions. Early fragmentation of peptide/metal complexes used CID to dissociate the peptide backbone.^156,157^ In order to preserve the metal/peptide complex, low-energy CID was used in these studies; however, it remains a concern that the increase in internal energy could mobilise the metal cation sufficiently to migrate across the protein/peptide in the gas phase prior to backbone fragmentation. In some cases, weakly bound metal ions might be lost in CID, as discussed in Section 2.1.3. Since the development of ExD and photodissociation techniques, metal ions can be more reliably pinpointed on peptides and proteins. Metal ion binding sites have been identified on native peptide and protein monomers such as amyloid β,^158^ α-synuclein,^159,160^ and carbonic anhydrase^161^ with ECD, as well as native protein complexes such as alcohol dehydrogenase.^153,162^ Similarly, UVPD is also effective in determining metal binding sites as shown in model metalloproteins including staphylococcal nuclease, azurin, and calmodulin.^145^ Brodbelt and co-workers have also used this method to localise zinc ions within the insulin pentamer.^140^ Interestingly, in some of these studies it was shown that some CID fragments also retained the metal cation, and the pattern of *apo* and *holo* CID fragments was consistent with that from ECD – this was likely due to the 80-fold lower dielectric permittivity of vacuum compared to water strengthening the electrostatic protein–metal interactions. For the same reason, metal binding can sometimes survive monomer ejection, and a complex-down approach (see Section 2.2) has also been successfully applied to identify binding sites of endogenous metal cofactors of both soluble^36^ and membrane^163^ proteins.

**Fig. 6:**
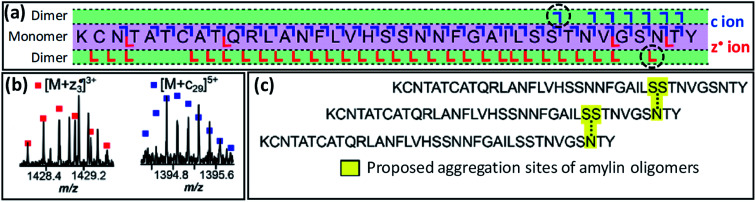
(a) ECD fragmentation map of the 7+ amylin dimer. The critical [M + *c*_29_] and [M + *z*_3_˙] fragments, shown in (b), are indicated by a black, dashed circle in this map. Panel (c) shows the proposed staggered structure of the oligomers. Adapted with permission from ref. 167. Copyright 2020 American Chemical Society.

Taking this a step further, native TD can provide information on binding interfaces between biological macromolecules. In this way, native TDMS has proven useful for understanding the quaternary structure of protein complexes as well as ‘lower’ levels of higher-order structure as discussed in Section 2.3.2. In 2009, Woods and co-workers used both ECD and ETD to investigate the residues involved in binding between small (<10 residues) acidic and basic peptides.^164^ Other pioneering work was performed by Langridge-Smith and co-workers in 2011, who used ECD to study the binding interface between the anterior gradient-2 protein and its hexapeptide ligand PTTIYY, concluding that binding involves the C-terminal part of the protein.^165^ Schneeberger and Breuker have used CID to determine the binding site of RNA on proteins, again taking advantage of the strengthening in the gas phase of electrostatic interactions to the point where they are occasionally able to survive backbone fragmentation.^166^ Recently, O'Connor and co-workers have shown that ECD fragmentation of oligomers of the amyloidogenic peptide amylin (implicated in type 2 diabetes) can provide information on the binding interface between monomers (see Fig. 6).^167^ Specifically, the observation of product ions consisting of an intact monomer noncovalently bound to either the three C-terminal residues (*z*_3_˙ fragment), or the 29 N-terminal residues (*c*_29_ fragment), led the authors to propose a model in which dimerisation occurs between Ser29 of the first, and Asn35 of the second monomer, in a staggered fashion.

#### 2.3.4 Native TD to study similarity and differences between solution-phase and gas-phase structures

As discussed in Section 1.2, there is an ongoing debate on how closely gas-phase structure reflects that in solution. Native TD has offered critical experimental data for understanding the evolving protein structures in the gas phase.^141,168^ Although some side chain rearrangements are expected from removal of solvent – for example, positively charged lysine side chains will rapidly form interactions with backbone carbonyl groups, as this intramolecular solvation is energetically highly favourable – the overall fold of protein complexes is generally believed to be kinetically trapped on the time scale of the MS analysis.^5^ However, inadvertent excessive gas-phase pre-activation of protein complexes – beyond what is needed for efficient desolvation – can lead to significant structural rearrangements, which can be manifested by changes in native TD spectra. Therefore, it is necessary to optimise MS tuning to reduce the amount of activation applied to a protein or protein complex. With little activation it seems that overall protein structure is preserved in the gas phase and can be readily probed for structural characteristics.

**Fig. 7:**
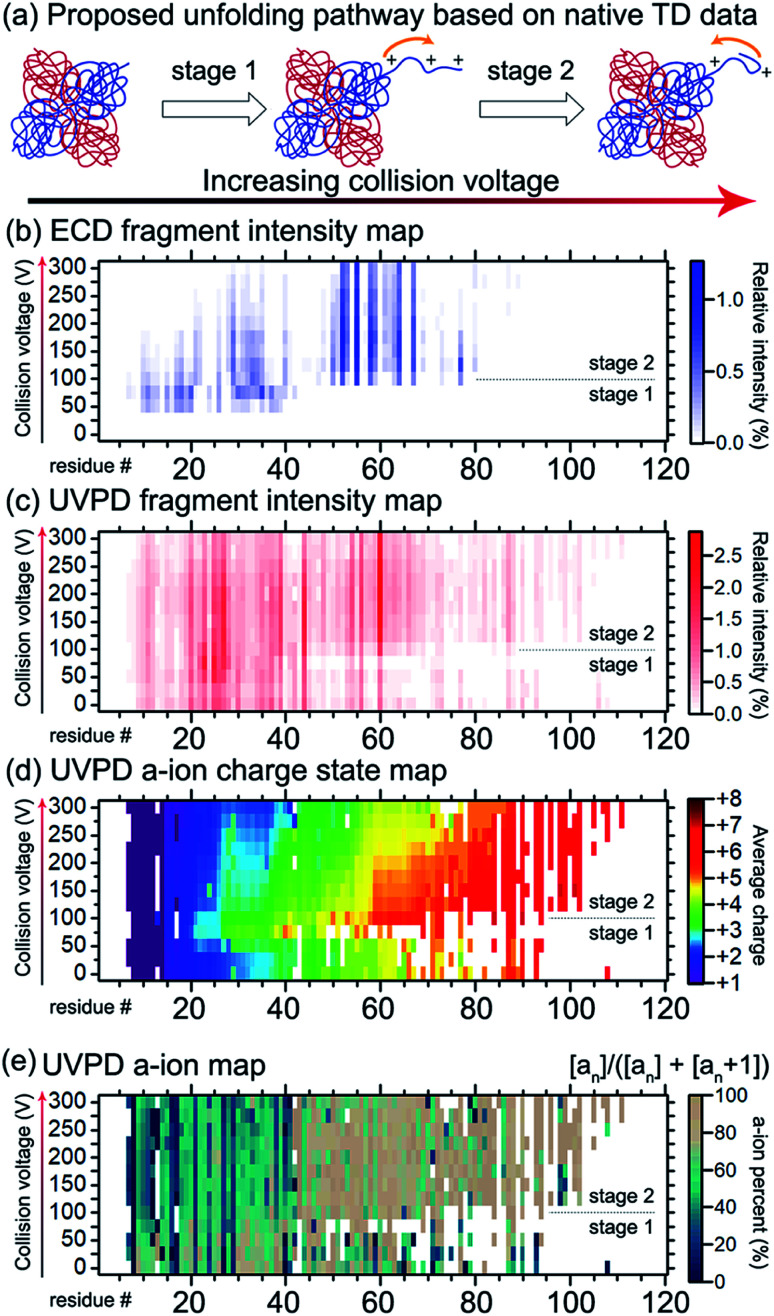
Probing the structural change of ADH by native TD. (a) The proposed two-stage structural changes to ADH in response to gas-phase activation. With increasing collision energy, the N-terminus unravels and then refolds. The mechanism is supported by the native TD data. (b) ECD N-terminal fragment intensity map along the first 120 residues of ADH. The vertical axis shows the increasing collision voltage. The horizontal axis shows the residue number. The colour represents the relative intensity, with the colour scale shown on the right. (c) UVPD N-terminal fragment intensity map with the same format as (b). (d) UVPD *a* ion charge state map. Same format was used as (b and c) except that the colour represents the intensity-weighted average charge state of *a* ions. (e) UVPD *a* ion map showing the percent intensity of *a* ions over the sum of *a* and *a* + 1 ion intensities. Similar to (b–d), the *a* ion map showed changes in the first 40 residues between the two stages with a collision voltage of *ca.* 100 V being the transition point. Adapted with permission from ref. 141. Copyright 2020 American Chemical Society.

Gross and co-workers demonstrated that native TD ECD fragments from the ADH tetramer reached deeper into the complex (starting from N-terminus into the core) as it unfolded with increasing activation in the ion source.^136^ The experimental data suggested that ADH unfolds through a ‘peeling an onion’ mechanism in which the N-terminus gradually unravels. Conceptually similar experiments reporting ECD and ETD of partially-unfolded haemoglobin were carried out in 2015 by the Gross,^169^ Loo,^170^ and Sobott^139^ groups, and recently ECD and ion mobility were performed on the same instrument, providing two orthogonal methods to probe unfolding of this tetramer.^171^ Recently, the unfolding of ADH was re-examined by native TD using both ECD and 193 nm UVPD on the same instrument, further indicating that the N-terminus unravels with increasing collision energy (see Fig. 7).^141^ Larger fragments reaching deeper into the core of the protein were detected for both ECD and 193 nm UVPD as higher in-source collision energy was used. Interestingly, subtle changes in spectral features indicated that unfolding was not a simple, gradual unravelling of N-terminal residues. The ECD fragments within the first 50 residues decreased in intensity with increasing collision energy, implying protection from fragmentation in the region. UVPD data showed changes in *a*/*a* + 1 ion ratio in the first 50 residues as well, suggesting secondary structural changes. In addition, charge movement was monitored by examining the charge sites based on charge states of UVPD *a* ions, as pioneered by Morrison and Brodbelt.^172^ Charge density first increased at the N-terminus, but then surprisingly decreased as the collision energy further increased. By combining all the available data, it was proposed that ADH underwent N-terminal unfolding followed by (partial) refolding.

The examples discussed here show the tremendous experimental detail that native TD offers for improving our understanding of gas-phase protein structure. Previous computational and theoretical studies have largely relied on complex-up experiments at the intact protein/subunit level, which do not provide information at the amino acid level. We anticipate that the integration of native TD and computational modelling will greatly enhance our understanding of critical factors that modulate gas-phase protein fragmentation and dissociation. The ability to perform gas-phase spectroscopy on intact proteins and complexes in MS is particularly exciting and allows a new level of structural information to be accessed. Action ion spectroscopy coupled to MS using free electron lasers with variable wavelengths is well-suited for structure analysis as demonstrated by work on small molecules, peptides, and oligonucleotides. This concept is based on the varying fragmentation efficiency of precursor ions in response to enhanced absorption at resonant photon energies. Infrared ion spectroscopy has already been demonstrated on small monomeric proteins.^32,173^ Circular dichroism was recently combined with MS for oligonucleotides.^174^ With ongoing development of advanced light sources coupled to native MS,^44,175^ we expect the possibility of performing spectroscopy analysis of native proteins and complexes for deep structural characterisation to emerge in the near future. The ability to mass-isolate species by MS-based methods could potentially transform structural biology research by offering complementary methods to study non-homogenous samples (*e.g.*, endogenous proteins isolated directly from biological matrices).

## 3. Conclusion and outlook

**Fig. 8:**
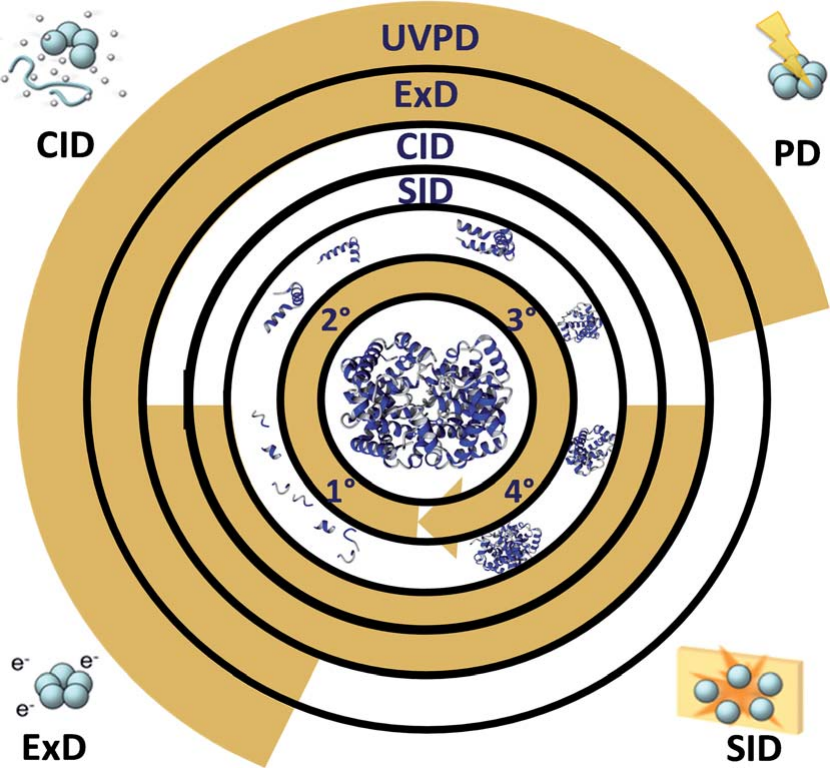
Common fragmentation methods for top-down fragmentation of native proteins and complexes. The centremost gold circle lists levels of structure (clockwise, from primary to quaternary). The gold shading in the ‘SID’, ‘CID’, ‘ExD’, and ‘UVPD’ circles indicates the structural levels each method is typically used to probe based on the existing literature. Cartoon representations of each method are shown in the four corners.

The biology of proteins must be considered through the lens of their ability to interact with one another, other biomolecules, and various cofactors and substrates. For this reason, complete knowledge of the primary structure, including post-translational modifications, is a necessary but insufficient condition to understand protein function. Recent methodological developments have enabled direct probing of the higher-order structure of native proteins and complexes, including membrane proteins, by mass spectrometry. At the lowest level of information, the mass and overall size of a complex can be measured using ion mobility-mass spectrometry; however, by combining native ionisation with top-down fragmentation, a range of workflows become accessible. These allow probing of subunit connectivity (complex-up), efficient ligand identification and monomer sequencing (complex-down), and elucidation of secondary and tertiary structure (native TD). The most commonly used ion activation methods in these experiments are summarised in Fig. 8, along with the structural levels they are typically used to probe. These recent developments in MS technology are complemented by advances in native separation strategies. These will become essential, as the future of this field will include analysis of native proteins from complex systems such as cells and tissues. We expect both separation and MS methods to see further development in the near future to address even larger assemblies and heterogeneous mixtures. Ongoing development of TDMS software will soon enable identification of all peaks present in native MS, complex-down, and native TDMS spectra. Furthermore, the top-down MS field has historically been to some extent dominated by a relatively small group of laboratories, based on the sophisticated equipment (in many cases custom modified mass spectrometers) and expertise required. However, the multinational Consortium for Top-Down Proteomics has launched an initiative aimed at making these workflows accessible to more labs. As instruments, methods, and knowledge become more accessible, we expect to see these methods being adopted by significantly more researchers in the next few years.

## Conflicts of interest

There are no conflicts to declare.
